# Predictors of preoperative anxiety among surgical patients in Jimma University Specialized Teaching Hospital, South Western Ethiopia

**DOI:** 10.1186/1471-2482-14-67

**Published:** 2014-09-05

**Authors:** Seifu Nigussie, Tefera Belachew, Wadu Wolancho

**Affiliations:** 1 Department of Nursing, College of Medical and Health Sciences, Samara University, Samara, Ethiopia; 2 Professor of Nutrition, Department of Population and Family Health, College of Public Health and Medical Sciences, Jimma University, Jimma, Ethiopia; 3 Department of Nursing, College of Public Health and Medical Sciences, Jimma University, Jimma, Ethiopia

**Keywords:** Anxiety, Ethiopia, Patients, Preoperative, State and trait anxiety inventory scale, Surgery

## Abstract

**Background:**

Hospitalization and surgery are critical negative life events that lead to the experience of considerable anxiety in patients. Patients may perceive the day of surgery as the biggest and the most threatening day in their lives. There is paucity of information on predictors of anxiety in the current study area. The main objective of this study is to assess predictors of preoperative anxiety among patients scheduled for surgery in Jimma University Specialized Teaching Hospital.

**Methods:**

A facility based cross-sectional study was conducted using quantitative data collection technique in Jimma University Specialized Teaching Hospital from February 13 to April 13, 2012 on 239 patients scheduled for surgery. The data were collected by five trained diploma nurses using structured interviewer administered questionnaires that were prepared based on state trait anxiety inventory measurement scale. The quantitative data were entered into SPSS for windows version 16. 0 and descriptive, simple and multiple linear regression analyses were performed.

**Results:**

A total of 239 patients were enrolled in the study with a response rate of 93.0%. Their mean age was 42.7 ± 1.8 years (range 16 to 85 years). Nearly over half 53.6% were females, while 48.1% illiterate, 72.4% Oromo and 56.5% were Muslim followers. Significant preoperative anxiety was seen in 70.3% patients. The most common factors that lead to anxiety were fear of death 38.1% and fear of unknown origin 24.3% and the most common strategy mentioned by patient in reducing anxiety were talking to other patient 79.8% and religious belief.

**Conclusions:**

In the present study, two third 70.3% of preoperative patients had anxiety. Factors which were positively correlated with anxiety were trait anxiety, single and divorced, time of operation and income. Factors which were shown to reduce anxiety were preoperative anxiety related information provision and afternoon operation. Health professionals working in the hospital should provide anxiety related information for patients.

## Background

Major life changes are among factors that cause anxiety, and one of these changes is surgery. Hospitalization, regardless of disease, is known to provoke anxiety in the patient admitted for surgery. If unrecognized, prolonged anxiety creates stress which may subsequently harm the patient and delay recovery [1-4]. Preoperative anxiety is a challenging concept in the preoperative care of patients. Most patients awaiting elective surgery experience anxiety and it is widely accepted as an expected response [5].

Patients may perceive the day of surgery as the biggest and the most threatening day in their lives. The degree to which each patient manifests anxiety related to future experiences depends on many factors. These include age, gender, type and extent of the proposed surgery, previous surgical experience, and personal susceptibility to stressful situations [6]. The reported incidence of preoperative anxiety ranges from 60% to 92% in unselected surgical patients and also varies among different surgical groups [7,8].

Preoperative anxiety is associated with problems such as difficult venous access, delayed jaw relaxation and coughing during induction of anesthesia, autonomic fluctuations, and increased anesthetic requirement. It has also been correlated with increased pain, nausea and vomiting in the postoperative period, prolonged recovery and increased risk for infection [9-13].

Many patients experience substantial anxiety before operation, and this is reported to affect 60–80% of surgical patients. Increased anxiety before surgery is associated with path physiological responses such as hypertension and dysrhythmias and may cause patients to refuse planned surgery [6,10].

The measurement of preoperative anxiety in modern elective surgery is becoming very difficult to administer, mainly due to the imposed time restrictions [14]. In a study carried out in Turkey (2011) on patients undergoing surgery, most of the patients awaiting surgery experienced high levels of preoperative anxiety. The anxiety scores were found to be higher among females than males. Results suggest that individuals with a high level of education may more accurately estimate the risk of surgery; however, individuals with low levels of education may fear the unknown and therefore have high levels of anxiety. There was no association between age and anxiety. Patients undergoing moderate level surgery had higher anxiety levels than patients that had major operations [1].

The most common reason for anxiety was the possibility of surgery being postponed (69.6%), followed by fear that mistakes may be made during the surgical operation resulting in harm to the patient (64%), fear of not receiving enough attention from care givers (63.2%) and fear of “not waking up” after surgery (58.4%). The respondents were least worried about having post-operative nausea and vomiting (8%). Only 27.1% male participants and 40.9% of the female participants were significantly anxious. But the gender difference was not statistically significant [15].

Result of study done by Atanassova M. (2009) showed the presence of a high degree of state trait anxiety-state (STAI – S) (78.1%) and state trait anxiety-trait (STAI – T) (62.5%) preoperative anxiety. Patients aged 50–59 and > 60 years, with previous anesthetic experience, less educated and non-smokers had higher frequency of preoperative anxiety [16].

Over all 62% patients had significant preoperative anxiety (having STAI-S) scores of 44 and above) [3]. Females were found to be more anxious than males. As age increased, the anxiety frequency decreased. There was significant correlation between level of education and preoperative anxiety [17,18].

Change of environment, waiting time of surgery, postoperative pain, concern about family, fear of one’s life, nil per mouth, blood transfusion, fear of unknown, harm from doctor/nurse mistake, getting stuck with needles and awareness during surgery were the significant factors responsible for increase pre-operative anxiety in women as compared to males [19,20].

The results showed that the majority (66.7%) of patients had moderate anxiety. Regarding correlation of religiosity and anxiety, the results did not show a significant relationship, but there was a reverse correlation between them. Patients with low religiosity had moderate to severe anxiety [21].

The prevalence of high state-anxiety was 23.99%. More years in school, medium surgery, history of cancer, history of smoking and negative perception of the future were associated with high preoperative anxiety. Previous surgery was associated with lower risk for preoperative state-anxiety, indicated by a negative coefficient [22].

Strategies to manage anxiety commonly used by the respondents included pharmacological interventions to relieve anxiety and pain and non-pharmacological interventions that involve information, communication, and stress reduction. Although these strategies are useful, they may not effectively reduce anxiety for all patients [8,23,24].

The research done in Hong Kong Chinese day patients suggest that providing self-selected music to day procedure patients in the preprocedure period assists in the reduction of physiological parameters and anxiety, yet, a relaxing environment can assist in the reduction of physiological parameters [25-27]. Little is known about preoperative anxiety; understanding this will be important to inform policies and strategies in provision of preoperative anxiety reduction care services. Therefore, the objective of this study was to assess the predictors of preoperative anxiety in JUSTH of Jimma town, south western Ethiopia. Prevalence of preoperative anxiety, factors responsible for preoperative anxiety and predictors of preoperative anxiety were the major areas that were presented in this study. The result of the study will provide information concerning preoperative anxiety to local as well as national and international policy maker, health professional, governmental and nongovernmental organization that work on presurgical anxiety and significant other so that it will help them to take appropriate action.

## Methods

### Study area and period

The study was conducted from February 13 to April 13, 2012 in Jimma University Specialized and Teaching Hospital found in Jimma town. The town is located 357 Kilometers South West of Addis Ababa with a total population of the town 128,330 (2007 central statistical agency (CSA) report). Jimma University Specialized and Teaching Hospital (JUSTH) is one of the oldest public hospitals in the country. It was established in 1937 by Italian conquerors for the service of their soldiers. At first, it was called “Ras Desta Damtew Hospital” after the name of an Ethiopian patriot during Italian career. It provides specialized health services through its 9 medical and other clinical and diagnostic departments for approximately 9,000 inpatients and 80,000 outpatients each year with bed capacity of 450 (from these there are 125 surgical beds in three surgical section) and a total of more than 700 staffs.

Currently, the hospital has more than 340 health professionals of different categories (30 specialists, 4 anesthetic nurses and 186 all other nurses) and 258 supporting staffs. Out of these health professionals, 190 are nurses (145 Diploma, 34 BSc.N, and 1 MSc.N), 50 are physicians, 20 are medical laboratory and laboratory technicians,17 are pharmacist and pharmacy technicians, 10 are radiographers and radiologists, 4 Health assistant, 2 Sanitarian and 15 are patient registration clerks and others like physical therapists. Operation room (OR) unit is one of the units of JUSH. In the hospital different surgery were performed such as thyroidectomy, mastectomy, colostomy, herniorraphy, hemoriedectomy, laparatomy, and etc.

### Study design

A facility based cross-sectional study design was used to assess the predictors of preoperative anxiety among surgical patients in JUSTH.

### Population

The source population was all clients/patients diagnosed and scheduled for surgery in JUSTH. The study subjects were all selected clients/patients who were diagnosed and scheduled for surgery in JUSTH during the study period. The study included all patients 15 and above years and those patients able to comprehend and can communicate and excluded patient diagnosed with any types of anxiety disorder such as phobic, panic, generalized, obsessive compulsive etc. and patients taking anti-anxiety or anti-depressant medications such as benzodiazepines, tricyclic antidepressants, selective serotonin inhibitors, monoamine oxidase inhibitors and lithium.

### Sampling method

A formula for estimation of single population proportion was used to calculate the sample size. Since there is no previous study on predictors of preoperative anxiety, to obtain optimum sample size, calculation was done using the assumption of proportion (**p**) of surgical patients facing anxiety 50%, with 95% CI, 5% marginal error (where **n** is minimum sample size, **Z** is value of standard normal variable at 95% confidence interval, **p** is maximum expected proportion which is 50% and **d** is marginal error which is 5%).

$$\begin{array}{c} n=\frac{Z^{2}\alpha /2\;P\;\left(1‒P\right)}{d^{2}}\\ =\frac{{\left(1.96\right)}^{2}*0.5*0.5}{{\left(0.05\right)}^{2}}\\ =384\;\mathrm{surgical}\;\mathrm{patents} \end{array}$$

Then correction formula was used as the number of patients scheduled for surgery is < 10, 000:

$$\begin{array}{c} \mathrm{nf}=\frac{n}{1+\frac{n}{N}}\\ =\frac{384}{1+\frac{384}{596}}=234\text{,} \end{array}$$

where N = 596 (Patients ≥ 15 years for whom surgery were performed in February and April of 2011 in JUSTH). Then considering 10% for non-response rate (23 individuals), the final sample size were 257 patients.

Systematic sampling technique was employed to select study participants. Dividing N/n (596/257 = 2), study subjects were selected every two patients until the sampled population fills.

### Data collection

#### Instrument

Data collection tools on preoperative anxiety were adapted and modified from validated questionnaire used on other study [28-30]. The questions and statements were grouped and arranged according to the particular objectives that it can address based on experts comments. Level of anxiety and need for information about surgery and/or anesthesia were assessed with the State Trait Anxiety Inventory Scale (STAIS) [31]. Reliability and validity of the STAI are well reported (Cronbach’s alpha = 0.896).The STAI is suitable for individuals who are 15 years old and older. The STAI Form Y is the definitive instrument for measuring anxiety in adults. It clearly differentiates between the temporary condition of “state anxiety” and the more general and long-standing quality of “trait anxiety”. It helps professionals distinguish between a client’s feelings of anxiety and depression. The inventory’s simplicity makes it ideal for evaluating individuals with lower educational backgrounds. Adapted in more than forty languages, the STAI is the leading measure of personal anxiety worldwide. The STAI has forty questions with a range of four possible responses to each.

The State Anxiety Scale (STAI Form Y-1) consists of twenty statements that evaluate how the respondent feels “right now, at this moment”. The Trait Anxiety scale (STAI Form Y-2) consists of twenty statements that evaluate how the respondent feels “generally”. In responding to the S-Anxiety scale, the subjects choose the number that best describes the intensity of their feelings: (1) not at all, (2) somewhat, (3) moderately, (4) very much so. In responding to the T-Anxiety scale, subjects rate the frequency of their feelings on the following four-point scale: (1) almost never, (2) sometimes, (3) often and (4) almost always.

Each STAI item is given a weighted score of 1 to 4. A rating of 4 indicates the presence of high levels of anxiety for ten S-Anxiety items (#3, 4, 6, 7, 9, 12, 13, 14, 17 and 18) and eleven T-Anxiety items (#22, 24, 25, 28, 29, 31, 32, 35, 37, 38, and 40). A high rating indicates the absence of anxiety for the remaining ten S-Anxiety items and nine T-Anxiety items.

The scoring weights for the anxiety-present items are the same as the chosen numbers on the test form. The scoring weights for the anxiety-absent items are reversed. Scores for both the S-Anxiety and the T-Anxiety scales can vary from a minimum of 20 to a maximum of 80. The sum of the scores on all items constitutes the individual’s score.

Data was collected by five trained diploma nurses recruited from outside JUSTH staffs who are fluent speaker of Afan Oromo and Amharic through face to face interview method. The period of data collection was for two month (February 13, 2012 to April 13, 2012) and data was collected the night before the day of surgery. The trained data collectors collected the data using a structured interviewer administered Amharic and Afan Oromo version questionnaire. Supervisor and principal investigator closely supervised the process of data collection and verifies.

### Data analysis

The completed questionnaires were checked for inconsistencies and missed values. Incomplete questionnaires were excluded from the analysis. Before data entry, appropriate coding and editing was performed. After data entries checking of already entered data were performed and the analysis was performed using SPSS for windows version software package 16.0.

Simple and multiple linear regression analysis were used and assumptions such as linearity, normality, homoscedasticity and independence were considered. Common descriptive statistics were considered as per variables of interest. Statistical tests were performed at the level of significance of 5%. The results were summarized using tables and figures and presented with narrative descriptions.

### Ethical considerations

Ethical clearance letter was initially obtained from Jimma University College of Public Health and Medical Sciences Ethical Committee. Then written letter was submitted to JUSTH and Limmu Hospital clinical and nursing director to get permission. For participants greater than 18 years, written informed consent for participation in the study was obtained from participants and for participants in between 15–18 years consent was obtained from the parent or legal guardian of the patient. Further, study participants were briefed about the study by stating the main objective and any unclear points related to the study before the interview begun.

In addition, confidentiality of the information was assured and privacy of the study population was respected and kept as well. Moreover, to ensure confidentiality the name of respondents were not written on the consent form. Telling that his/her participation in the study is very important, every client to be interviewed was informed that he/she has a full right to discontinue the interview.

## Results

### Socio demographic characteristics

A total of 239 patients were enrolled in the study with a response rate of 93.0%. Their mean age was 42.7 ± 1.8 years (range 16 to 85 years).Out of the total respondents about 111 (53.6%) were females, 109 (45.6%) were between the age of 35 and 64 years, 173(72.4%) Oromo, 135(56.5%) Muslim, 188 (78.7%) married, 115(48.1%) illiterate and 114 (47.7%) were farmer.

Majority 64% of participants came from rural areas. Only 27 (11.3%) live alone while the rest 212 (88.7%) were living with other persons such as partner 95 (44.8%) and with their children’s 73 (34.4%). Forty participants were drug users and 10 participants had life insurance.

Only 75(31.4%) of study participants had information about previous surgeries. Seventy seven patients had an experience of previous hospitalization. Forty nine (20.5%) patients had undergone previous surgery under local 20(37.7%) and general anesthesia 33(62.3%) and two of them experienced surgery complications. Six patients had history of cancer.

Among 77(32.2%) of patients who were hospitalized previously, 53(68.8%) hospitalized once, 16(20.8%) twice, 4(5.2%) thrice and 4(5.2%) more than thrice. Regarding time of previous operation, from a total of forty nine patients who had experience of previous operation, 42(85.7) had operation within last five years, 5(10.2%) in between 5–10 years and 2(4.1%) greater than ten years. Majority 40(81.6%) had only once previous operation while 7(14.3%) twice and 2(4.1%) three times.

### Current health status of the study participants

**Figure 1:**
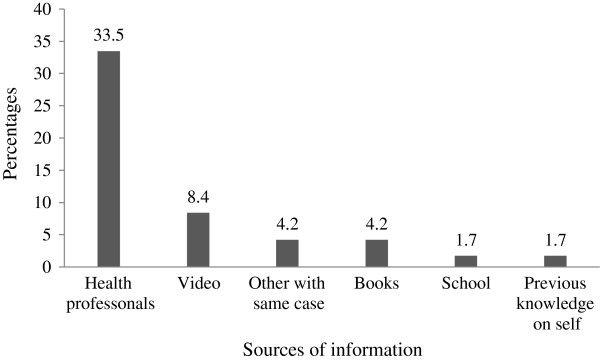
Sources of information for current surgery among patients attending surgical wards of JUSTH.

One hundred nine (45.6%) of study participants were scheduled for minor surgery while only 29(12.1%) were candidate for major surgery. From the total study participants, 182(76.2%) of them know their diagnosis, 125(52.3%) had the knowledge of type of surgery to be performed, only 45(18.8%) know type of anesthesia they are going to take and 17(37.8%) mentioned local anesthesia. One hundred forty three patients were in pain and 118(82.5%) experienced it before admission.The assessment of study participants information regarding their current surgery to be performed indicated that 110 (46%) of study participants had got information for their current surgery from different sources such as health professional 80(33.5%) and video 20(8.4%) (see Figure 1).

Larger proportion of study participants 156(65.3%) do not get information related to preoperative anxiety. Only 83(34.7%) get information on different topics such as about preoperative procedure 60(25.1%), expected recovery 24(10%) and pain expected 25(10.5%). Twenty five patients did not give informed consent. For those who gave informed consent 214(89.5%), majority 97(45.3%) simply sign, for 33 (15.4%) patients only the problems were briefed and 84 (39.3%) got information about the problem, procedure and anesthesia. Out of 239 patients anxiety assessment was done only for 42 (17.6%) patients.

### Factors responsible for preoperative anxiety

Observing different factors responsible for pre-operative anxiety showed that the most common factors were fear of death in 91(38.1%) patients, fear of unknown 58 (24.3%), financial loss 47(19.7%) and results of operation 46(19.2%). Only 4 (1.7%) patients were anxious because of awareness during surgery (see Table 1).

**Table 1:** Possible causes of anxiety among patients attending surgical wards of JUSTH

**Possibilities causing anxiety**	**Frequency**	**Percentages**	**Mean state anxiety score**
Fear of death	91	82.7	56.3 ± 8.2
Fear of unknown	58	52.7	57.8 ± 8.9
Financial loss due to hospitalization	47	42.7	54.7 ± 8.6
Results of operation	46	41.8	55.7 ± 7.9
Needing blood transfusion	43	39.1	57.4 ± 9.2
Concern about family	43	39.1	56.9 ± 6.9
Postoperative pain	43	39.1	55.7 ± 9.2
Change of environment	41	37.3	55.2 ± 7.7
Long waiting time for operation	40	36.4	54.9 ± 9.0
Unexpected events	39	35.5	55.2 ± 9.2
Being patient and coming to hospital	35	31.8	51.8 ± 8.9
Dependency	34	30.9	55.1 ± 8.7
Fear of physical disability	33	30	53.9 ± 8.7
Fear of complications	31	28.2	53.3. ± 7.3
Cancellations	31	28.2	54.6 ± 9.0
Alteration in body image	26	23.6	56.7 ± 7.5
Possible unknown diagnostic results	24	21.8	53.2 ± 6.6
Lack of information	18	16.4	51.3 ± 8.1
Nil per mouth	16	14.5	55.6 ± 1.0
Harm from doctor or nurse mistake	16	14.5	57.7 ± 8.8
Terminology and language used	16	14.5	53.8 ± 1.2
Sickness and vomiting	15	13.6	53.7 ± 8.3
Fear of not knowing what occurs while unconscious during anesthesia	14	12.7	52.8 ± 9.2
Concern about fellow patient	13	11.8	55.6 ± 9.2
Absence from work	13	11.8	55.7 ± 9.8
Inaccurate information	12	10.9	55.5 ± 8.9
Short waiting time for operation	10	9.1	56.1 ± 7.4
Getting stuck of needle	9	8.2	54.3 ± 1.2
Lack of knowledge of ward	9	8.2	51.3 ± 8.1
Not awakening from anesthesia	8	7.3	55 ± 1.2
Information from previous negative hospital experiences	8	7.3	55 ± 1.2
Familiarity with facilities	7	6.4	58 ± 7.1
Lack of recognition of staff	5	4.5	55.6 ± 6.7
Awareness during surgery	4	3.6	63.3 ± 2.5

Percentages do not add up to 100 as this is a result of multiple response questions.

### Prevalence of preoperative anxiety

Patients past (trait anxiety) and current (state anxiety) anxiety state were also assessed using State Anxiety Inventory scale. For state and trait anxiety there were 40 items (20 each) which were measured by Likert scale from 4. Regarding state anxiety item, 92 (38.5%) of patients scored moderate calm (see Table 2).

**Table 2:** STAI state anxiety score attending surgical wards of JUSTH

**S.No**	**Variables**	**Not at all**	**Somewhat**	**Moderately so**	**Very much so**
		**n (%)**	**n (%)**	**n (%)**	**n (%)**
1	I feel calm*	58 (24.3)	34 (14.2)	92 (38.5)	55 (23)
2	I feel secure*	72 (30.1)	49 (20.5)	67 (28)	51 (21.3)
3	I feel tense	66 (27.6)	85 (35.6)	56 (23.4)	32 (13.4)
4	I feel strained	125 (52.3)	54 (22.6)	27 (11.3)	33 (13.8)
5	I feel at ease*	28 (11.7)	55 (23)	74 (31)	82 (34.3)
6	I feel upset	141(59)	4 (20.1)	27 (11.3)	23 (9.6)
7	I am presently worried	72 (30.1)	68 (28.5)	64 (26.8)	35 (14.6)
8	I feel satisfied*	32 (13.4)	32 (13.4)	61 (25.5)	114 (47.7)
9	I feel frightened	52 (21.8)	53 (22.2)	67 (28)	67 (28)
10	I feel comfortable	53 (22.2)	68 (28.5)	61 (25.5)	57 (23.8)
11	I feel self-confident*	41 (17.2)	52 (21.8)	67 (28)	79 (33.1)
12	I feel nervous	136 (56.9)	52 (21.8)	38 (15.9)	13 (5.4)
13	I feel jittery	79 (33.1)	53 (22.2)	68 (28.5)	39 (16.3)
14	I feel indecisive	81 (33.9)	74 (31)	54 (22.6)	30 (12.6)
15	I am relaxed*	41 (17.2)	35 (14.6)	57 (23.8)	106 (44.4)
16	I feel content*	33 (13.8)	49 (20.5)	53 (22.2)	104 (43.5)
17	I am worried	55 (23)	50 (20.9)	67 (28)	67 (28)
18	I feel confused	62 (25.9)	61 (25.5)	56 (23.4)	60 (25.1)
19	I feel steady*	33 (13.8)	43 (18)	72 (30.1)	91 (38.1)
20	I feel pleasant*	47 (19.7)	27 (11.3)	64 (26.8)	101 (42.3)

*Positive questions which are reverse coded.

Over all 168(70.3%) patients had significant preoperative state anxiety (having S-STAI scores of 44 and above). The mean (±SD) scores for state-anxiety and trait-anxiety were 49.89 ± 11.2 and 47.79 ± 8.9, respectively. Trait anxiety which is a pattern of anxiety that can be considered a personality trait was also assessed. Over all 148(61.9%) patients had significant trait anxiety (having S-STAI scores of 44 and above). That is 61.9% of patient had anxious personality. Twelve patients (5%) patients were free of anxious personality while 15 (6.3%) had slight trait anxiety.

### Predictors of preoperative anxiety

The bivariate analysis revealed a significant association between preoperative state-anxiety and the following variables: marital status, educational status, occupation, income, substance use, pain experience, knowledge of type of anesthesia, preoperative anxiety related information provision, time of operation, extent of surgery to be performed and trait anxiety were associated with preoperative anxiety. The association was not observed when those variables entered into final model for educational status, occupation, substance use, pain experience, knowledge of type of anesthesia and extent of surgery to be performed. However, no significant associations were observed between preoperative state-anxiety and sex, age, residence, living arrangement, previous operation, previous hospitalizations, future self-perception, history of cancers and knowledge of type of surgery.

Multivariable linear regression was performed to determine the best linear combinations of marital status, educational status, occupation, time of operation, extent of operation, income, preoperative information provision, knowledge of type of anesthesia, substance use, pain experience and trait anxiety for predicting preoperative state-anxiety scores. This combinations of variables significantly predicted preoperative anxiety F (21, 217) = 14.985, p < 0.001, with marital status, time of operation, income, preoperative information, income and trait anxiety significantly contributing to the prediction. Being divorced contribute most to predicting preoperative anxiety, and that being single, having high income, getting preoperative anxiety related information, high trait anxiety and morning surgery also contribute to this prediction.

Being single had a preoperative state anxiety score of 5.288 (B = 5.288, 95% CI (2.149, 8.428) while being divorced had 5.629(B = 5.629, 95% CI (0.0531, 11.205) preoperative state anxiety score. For a unit increase in trait anxiety score preoperative state-anxiety score was increased by 0.719(B = 0.719, CI = (0.593, 0.846), p < 0.001). Similarly for a unit increase in income preoperative state-anxiety score was increased by 0.002 (B =0.002, CI = (0.001, 0.004), p < 0.001). Preoperative anxiety related information provision was negatively associated with preoperative state anxiety. That means the more anxiety related information provision the less preoperative state anxiety (B = −2.337, CI = (−4.656, −0.018), p = 0.048). Time of operation was also negatively associated with preoperative state anxiety. Those patients who will be performed operation in the afternoon on the average 2.8 less anxiety compared to those expected operation in morning (B = −2.770, CI = (−4.906, −0.633), p = 0.011) (see Table 3).

**Table 3:** Multivariable linear regression model showing predictors of preoperative anxiety

**Variables entered into model**	**B**	**SE**	**P**	**95% CI**
Marital status				
**Single**	5.288	1.593	0.001*	**(2.149,8.428)**
**Divorced**	5.629	2.829	0.048*	**(0.053,11.205)**
Widowed	−2.672	3.314	0.421	(−9.203, 3.859)
Educational status				
Read and write	−0.440	1.828	0.810	(−4.042, 3.163)
Grade1- 6	0.859	1.771	0.628	(−2.632, 4.350)
Grade 7-8	−3.395	2.170	0.119	(−7.673, 0.882)
Grade 9-12	0.699	1.962	0.722	(−3.168, 4.565)
College and above	2.389	2.137	0.265	(−1.823, 6.601)
Occupation				
Government worker	−2.190	2.125	0.304	(−6.379, 1.999)
Private worker	0.699	1.440	0.628	(−2.139, 3.537)
House wife	1.869	1.960	0.342	(−1.995, 5.732)
Student	0.318	2.753	0.908	(−5.109, 5.745)
**Income**	0.002	0.001	0.001*	**(0.001, 0.004)**
Substance use				
No	−1.769	1.546	0.254	(−4.816, 1.279)
**Trait anxiety**	0.719	.064	0.000*	**(0.593, 0.846)**
Presence of pain				
Yes	0.334	1.064	0.754	(−1.762, 2.431)
Time of operation				
**Afternoon**	−2.770	1.084	0.011*	**(−4.906, −0.633)**
Preoperative information provision				
**No**	−2.337	1.176	0.048*	**(−4.656, −0.018)**
Knowledge of type of anaesthesia				
No	1.278	1.450	0.379	(−1.581, 4.137)
Extent of surgery to be performed				
Medium	−0.231	1.084	0.831	(−2.368, 1.906)
Major	1.683	1.704	0.324	(−1.675, 5.041)

*Variables which are candidate for multiple linear regression (<0.05) were included in the model. Note: R^2^ = 0.592, F (21,217) =14.985, p < 0.001.

Statistical significant (p < 0.05).

Married, illiterate, farmer, yes (for substance use, preoperative information, and knowledge of type of anesthesia), morning, absence of pain and minor were used as reference category.

## Discussions

Anxiety is a common response to stress and is present in patients scheduled for surgery. As with pain, assessment of presence of anxiety and quantifying is difficult. In the past, various investigators described different methods for quantifying anxiety. In broad terms these were either self-reporting questionnaires or objective tools which measured the activity of stress hormones. All these methods have their limitations. Among the available tools, State Trait Anxiety Inventory questionnaire (STAI) is currently taken as a gold standard because it has shown consistent results in different population and ethnic groups in assessing anxiety and is available in various languages [3,30,32]. Most patients awaiting elective surgery experience anxiety and it is widely accepted as an expected response to this situation [1].

Over all frequency of preoperative anxiety in the study was 70.3% as suggested by STAI score of more than 44. The findings of this study showed that most of the patients awaiting elective surgery experienced high levels of preoperative anxiety. This is not in line with the finding of the study done in Pakistan. This difference could be due to the fact that Pakistan is relatively developed country than Ethiopia so that patients had access to information regarding their surgery and they also ask health professionals [3]. But it is in line with the finding reported in the study done in Turkey [1].

Study done in Pimpri, India, teaching hospital indicated that patients who were well informed about the surgical procedure in advance had significantly less preoperative anxiety than those unaware of the procedure [33]. This is consistent finding with the present study. This may suggest that information plays a great role in reduction of preoperative anxiety.

The most common reason for anxiety in Port Harcourt Teaching Hospital, city in southeastern Nigeria, was the possibility of surgery being postponed (69.6%), followed by fear that mistakes may be made during the surgical operation resulting in harm to the patient (64%), fear of not receiving enough attention from care givers (63.2%) and fear of “not waking up” after surgery (58.4%). The respondents were least worried about having post-operative nausea and vomiting (8%) [15]. This is inconsistent with the present study. The reason could be due to difference in sociocultural, socio economic and area difference.

Patients awaiting surgical procedures have various reasons for their preoperative anxiety. In this study to determine the different aspects of preoperative anxiety; patients were offered a list of different causes. Preoperative anxiety is related to fear of the unknown, unfamiliar place, loss of control of situation, and fear of death. Interestingly, fear of death was the patient’s greatest concern followed by fear of unknown. Results in Pakistan showed that the patient’s greatest concern was concern about family followed by fear of complications, while in this study they ranked sixth and fourteenth respectively [5]. This difference could be as the researcher mentioned Pakistan was the very family oriented local setup, where the joint family system is still intact to a great extent, and where family values are quite important. Here in our study area although the population concern for family since the community view the health professional’s ability as low and there were also not as such necessary equipment, the patients had in mind the probability of death. Interestingly, awareness during surgery was the least concern for our patients which was consistent with Pakistan but not with Nigeria [5,15].

There are several risk factors for preoperative anxiety. These include history of cancer, psychiatric disorders, self-perception, depression, trait-anxiety level, pain, history of smoking, extent of the proposed surgery, female gender, level of education, and physical status according to ASA [1]. In this study there was a significantly higher level of anxiety in single and divorced. Similar finding have also been reported in the literature [2] while some other investigators demonstrated the lack of marital status effect [34].

The percentage of participants that were found to be anxious in this study was more in females than in males, but this was not statistically significant. Some studies have shown that females experience more preoperative anxiety than males [3,7], whereas other workers found that gender was not a determinant of preoperative anxiety [15]. A further study on this subject with a larger sample size is suggested. The proportion of participants with preoperative anxiety in this study appeared to increase with increasing level of education and decrease with increasing age, but this also was not statistically significant. This was in conformity with results of previous studies [15].

Patients who had previous surgical experience would be less anxious than patients waiting for surgery for the first time. Contrary to this, in our study no significant difference was noted [5].

Trait anxiety which is a pattern of anxiety that can be considered a personality trait was also assessed. High state-anxiety levels indicate high levels of anxiety at the moment of evaluation and high trait-anxiety levels indicate an anxious personality. There was a significant correlation between this anxious personality and preoperative state anxiety in the study area [22,24].

There was an inverse correlation between the preoperative anxiety scores and preoperative information provision. These findings illustrate that as information provision increased, preoperative state anxiety decreased. These observations are supported by some studies [29] but not by other studies [35]. Another factor which was negatively correlated with anxiety was time of operation.

In this study, increasing income was associated with an increased level of preoperative anxiety.

There was no difference in state anxiety levels between males and females. Some previous studies support this finding [36] while others found that gender was a determinant of preoperative anxiety and females were more anxious than males [3]. Similarly education level did not influence the state anxiety level in this study.

Level of religiosity as a predictor of preoperative anxiety was not done. But some literatures get significant association while others do not [21]. Study done in Ankara, Turkey indicated that religion is commonly used as a way of coping with stress caused by health problems. This is in line with this study. Patients were asked their way of coping strategy from anxiety and they forwarded music which was also supported by others [11,25,26,37-39].

The findings of this study suggest that practice of giving preoperative information can reduce patient anxiety. Few patients were informed about anesthesia, but this finding did not affect state-anxiety levels. Knowledge about anesthesia or diagnosis did not influence state-anxiety levels. The percentage of patients informed about anesthesia was low and might be considered a subject to be addressed (surgery information) [36,40].

## Conclusions

In the presented study, the prevalence of preoperative anxiety is high and no steps were taken to reduce this. The most common factors that make patients to suffer from anxiety were fear of death, fear of unknown origin, financial loss and results of operation.

Factors which were positively correlated with anxiety were STAI trait, single and divorced, time of operation and income and factors which were shown to reduce anxiety were preoperative anxiety related information provision and afternoon operation. The major coping mechanisms that were forwarded by patients were talking their anxiety to other patients and giving all things for God or Allah. The most common effective way of reducing anxiety mentioned by patients was religious belief. Consent form, anxiety assessment and intervention for anxiety were not got attention by health professionals.

## Abbreviations

ASA: American Society of Anesthesiology; BP: Blood pressure; BScN: Bachelor of Science in Nursing; CI: Confidence interval; CSA: Central Statistical Agency; HPA: Hypothalamus pituitary adrenal; JUSTH: Jimma University Specialized Teaching Hospital; OR: Operation room; PR: Pulse rate; SD: Standard deviation; SPSS: Statistical Packages for Social Science; STAIS: State trait anxiety inventory scale.

## Competing interests

The authors declare that they have no competing interests.

## Authors’ contributions

All authors (SN, TB and WW) participated to the design of the study and the interpretation of data. SN conceived of the study and performed the data analysis and drafted the manuscript. All other authors (TB and WW) critically revised the manuscript and have approved the final version. All authors read and approved the final manuscript.

## Pre-publication history

The pre-publication history for this paper can be accessed here:

http://www.biomedcentral.com/1471-2482/14/67/prepub
